# Six-Axis Force Torque Sensor Model-Based In Situ Calibration Method and Its Impact in Floating-Based Robot Dynamic Performance †

**DOI:** 10.3390/s19245521

**Published:** 2019-12-13

**Authors:** Francisco Javier Andrade Chavez, Silvio Traversaro, Daniele Pucci

**Affiliations:** Dynamic Interaction Control Lab at Istituto Italiano di Tecnologia, 16163 Genova, Italy; silvio.traversaro@iit.it (S.T.); daniele.pucci@iit.it (D.P.)

**Keywords:** force torque sensors, in situ calibration, robot dynamic performance

## Abstract

A crucial part of dynamic motions is the interaction with other objects or the environment. Floating base robots have yet to perform these motions repeatably and reliably. Force torque sensors are able to provide the full description of a contact. Despite that, their use beyond a simple threshold logic is not widespread in floating base robots. Force torque sensors might change performance when mounted, which is why in situ calibration methods can improve the performance of robots by ensuring better force torque measurements. The Model-Based in situ calibration method with temperature compensation has shown promising results in improving FT sensor measurements. There are two main goals for this paper. The first is to facilitate the use and understanding of the method by providing guidelines that show their usefulness through experimental results. Then the impact of having better FT measurements with no temperature drift are demonstrated by proving that the offset estimated with this method is still useful days and even a month from the time of estimation. The effect of this is showcased by comparing the sensor response with different offsets simultaneously during real robot experiments. Furthermore, quantitative results of the improvement in dynamic behaviors due to the in situ calibration are shown. Finally, we show how using better FT measurements as feedback in low and high level controllers can impact the performance of floating base robots during dynamic motions. Experiments were performed on the floating base robot iCub.

## 1. Introduction

Robots are expected to perform highly dynamical motions. Being able to perform these motions repeatably and reliably is an active research topic. A crucial part of these motions is the interaction of the robot with other objects or the environment. Whenever an interaction happens there exist an exchange of forces. Therefore, knowledge of the forces exchanged at contacts is a fundamental part of endowing robots with the ability to perform dynamical motions. The six-axis force-torque (FT) sensors convey a complete information of a contact force by providing measurements of the three axes of forces and three axes of torques. Robots with their base fixed to the ground have been using FT sensors to measure contact forces since a long time [1,2,3]. Despite being common sensors in many floating base platforms [4,5,6,7,8,9], their potential use in floating base robots has not been full documented. One of the reasons is that the reliability of this sensors may decrease after mounting them [10,11,12,13]. The Model-Based In Situ Calibration Method has shown promising results in the improvement of the reliability of force torque sensors [14]. By extending its benefits and showcasing the improvement of floating base robots performance with this method, we aim to encourage the use of this sensor in these types of platforms in more effective ways.

The most common phenomena used in FT sensors to measure forces is the change in resistance of silicon due to strain [15]. In more technical words, the piezoresistive response to strain of semiconductor material. This material also changes resistance with temperature. Because of this, depending on the calibration procedure, the sensor might suffer from temperature drift [14,16,17]. This is the undesired change of measurement due to changes in temperature. Methods to deal with temperature compensation such as an array of strain gauges in a Wheatstone Bridge configuration are ineffective in these sensors due to the inner structure of FT sensors.

Typically, the offset in FT sensors is removed before use. Commonly floating base robots use this sensor to detect and measure contacts. Thus, the time of collision is unknown *a priori*. Removing the offset after the sensor has established contact would make the value measured by the FT sensor incorrectly. Therefore, minimizing the effect of drift in the FT sensors can improve the reliability of the sensor in floating base robots.

The typical calibration procedure considers, first, identifying the offset when no load is applied on the sensor. Then, carefully placing some weights in specific positions to have well known forces and torques in order to span the space of the sensor. To resolve the coupling effects, it is necessary to have calibration points with as many orientations of the force vector as possible based on the sensor’s coordinate system. The calibration data should ideally be a representative dataset of what the sensor will be subjected to.

Due to the nature of the technology used, solving with least squares remains the most popular [18]. Calibrations of these sensors are known to loose effectiveness over time. Leading companies for FT sensors [19,20] recommend to calibrate the sensors at least once a year. This normally implies that the sensor must be unmounted, sent back to them and then mounted again.

The most common calibration procedure is a quasi-static calibration of the sensor. Calibration procedures can be classified into ex situ when the sensor is calibrated in a place different where is used, and in situ if it is calibrated on the place it will be used.

Ex situ calibration is usually performed using specialized structures [1,21,22,23] or relying on previously calibrated sensors [24,25,26,27]. The latter has the disadvantage of trusting on the calibration of another sensor which might not be perfect. The in situ methods allow to perform the calibration in the sensor’s final destination, avoiding the decreases in performance that arise from mounting and removing the sensors from its working structure. Some in situ calibration methods have relied on other FT sensors [27,28]. Others exploit known relationships between some quantities such as joint torques [29] or acceleration measurements [10] to obtain the reference forces. Some calibrate the sensor mounted in their final position by designing a calibration bench that accommodates the sensor and the mounted structure [30]. This requires the design of a particular structure and the mounting and dismounting of the whole part to calibrate one sensor. Six-dimensional force/torque sensors can be calibrated based on the shape from motion method with complex algorithm. This requires the use of thre different sets of weights and a minimal setup with a fixed pulley. It requires a calibration of the sensor three times per load, so in total, nine datasets [31]. In all of these methods, the effect of temperature is either not considered or carefully controlled when calibrating without accounting for changes in the working conditions. Other methods exploit the encoders and the model of the robot to provide the reference forces and torques [11]. This method was successfully improved to account for temperature by considering temperature effects on the sensor as linear [14].

There are two main contributions of this paper. The first is to provide useful tips to better exploit the Model-Based in situ calibration method with temperature compensation in a simple way. The other is to showcase the impact of the improved measurements obtained with this calibration in the dynamic performance of floating base robots.

The paper is structured as follows: in Section 2, the in situ calibration method is detailed. It also contains a description of the robotic platform and the ways the sensor information is used in such a platform. Section 3 contains the useful tips to better exploit the Model Based in situ calibration method with temperature compensation in a simple way and shows a way to exploit the generic formulation to simultaneously estimate offset and calibration matrix. We also describe two new types of datasets with the aim to discuss how to understand sensor excitation easily. A simple non-intrusive method to exploit the resulting calibration is described. A description of the constant offset hypothesis can be found as well. Section 4 details how the experiments were setup, the datasets used and how the results were validated. In Section 5, the results are used to verify the usefulness of the tips, the constant offset hypothesis and improvements in floating-based robot performance when using the Model-based in situ calibration method with temperature compensation. Conclusions can be found in Section 6.

## 2. Background

In this section, the developed in situ calibration method is described in detail. Starting from the general mathematical model of the FT sensor to the problem statement that is solved through the Model-Based in situ calibration method.

### 2.1. Mathematical Model of FT Sensors

There are two physical laws at play in strain gauge force sensors. One is common to all kinds. It is the relationship between the deformation of a spring and forces, it is the Hooke’s law of elasticity.

(1) f=k∆x,

where *f* is the force value in N, *k* is a constant of the material $N\;m^{-1}$ and ∆x is the displacement (or strain) in meter. It is valid as long as the material does not reach plastic deformation. Another definition of Hooke’s Law is the relationship between engineering stress and engineering strain for elastic deformation [32]. Stress σ (Pa) is expressed in terms of force applied to a certain cross-sectional area *A* ($m^{2}$) of an object,

(2) σ=f/A.

For FT sensors *A* is the cross-section of an internal beam of the FT sensor. Strain ϵ is the deformation of a physical body under the action of applied forces. It has no units. Strain is calculated as

(3) $$\epsilon =\frac{l_{i}-l_{0}}{l_{0}}=\frac{∆l}{l_{0}},$$

Using the other definition of Hooke’s Law the relationship between stress and strain is:

(4) σ=Eϵ

where E is the modulus of elasticity of the material and depends on the kind of stress and strain applied to it. The other principle depends on the type of sensing technology. For semiconductor strain gauges it is the piesoresistive effect. The model is the linear function [15]:

(5) $$R=R_{o}\left(1+S_{\epsilon }\epsilon \right),$$

where *R* is the resistance value in Ω, ϵ is the strain, $S_{\epsilon }$ is the gauge factor of the conductor, $R_{o}$ is the resistance with no stress applied in Ω.

Combining both physical effects gives the following transfer function:

(6) $$R=R_{o}\left(1+S_{\epsilon }\frac{f}{EA}\right)=R_{o}\left(1+S_{\epsilon }\frac{f}{k}\right)$$

Therefore the most used model for predicting the force-torque from the raw strain gauges measurements of the sensor is a linear model. The inverse function is:

(7) $$f=\frac{(R-R_{0})k}{S_{\epsilon }R_{0}}=\frac{Rk}{S_{\epsilon }R_{0}}-\frac{k}{S_{\epsilon }}=cR-O,$$

where

$$\begin{array}{cc} c= & \frac{k}{S_{\epsilon }R_{0}}\\ O= & \frac{k}{S_{\epsilon }}. \end{array}$$

Considering possible errors during the calibration procedure, linear regression is the most suitable approximation function. Multi-axis force-torque sensors usually contain multiple strain gauges, each of them can be seen as a separate sensor. Because of this, multiple regression is a valid option for this kind of sensor. Therefore, each force axis will be calibrated using the information from all strain gauges,

(8) $$f_{i}=c_{1}R_{1}-O_{1}+c_{2}R_{2}-O_{2}+\ldots c_{m}R_{m}-O_{m},$$

where $f_{i}$ is the force in the i-th axis in N or N m depending on the axis, $R_{m}$ is the digital response of the m-th strain gauge in bit counts, $c_{m}$ is the slope of the linear model of the m-th strain gauge in N/bit and $O_{m}$ is the bias of the m-th strain gauge. The orientation of $f_{i}$ with regards to the m-th strain gauge will change the value of *k* required. It depends on the strain being normal, shear or a combination of both. As a result, the array of $c_{m}$ coefficients $C_{m}$ and $O_{m}$ will be different for each *i*-th axis. Taking this in consideration, the approximation function for these sensors for all axes has the following form:

(9) f=Cr+o

where $f\in R^{6}$ are the 6D forces, $C\in R^{6\times m}$ is the calibration matrix in N/bit, $r\in R^{m}$ are the raw measurements (sensor’s response in bit counts) and $o\in R^{6}$ is the offset which is also a 6D force vector. Both the calibration matrix *C* and the offset *o* are unknown and need to be estimated. This formulation allows to calibrate all axes at the same time although they can be considered independent problems.

### 2.2. The Model-Based In Situ Calibration Method

Calibrating an FT in situ requires a dataset of samples $(r_{i},f_{i}),\;i=1\ldots N$ obtained from the sensor mounted on the robot. When no contact force is acting on the limb on which the FT sensor is mounted, the expected force-torque applied on the sensor can be computed using the robot model and the instantaneous joints position, velocity and acceleration [33,34,35]. Since reference forces $f_{i}$ are obtained this way, the method is named *Model Based In Situ Calibration*. The robot parameters are assumed to be known. There are three main components to this method. The first is to formulate the calibration problem decoupling the offset estimation problem from the calibration matrix estimation problem. The second one is to cast the calibration matrix estimation problem as a *regularized least square* problem, in which the regularization considers the known information of a previous calibration matrix. Lastly, having a way to consider other phenomena that might be creating some drift, the assumption considered is that other phenomena are also linear.

#### 2.2.1. Least Squares Solution

The multiple linear regression problem in Equation (9) can be solved using the least squares technique. The problem is stated as follows:

(10) $$\underset{C,o}{\text{arg min.}}\;\;\frac{1}{N}\sum_{i=1}^{N}{∥f_{i}-Cr_{i}-o∥}^{2}$$

where *N* is the number of data samples in the dataset. The offset is usually estimated separately from the calibration matrix. Because the offset can vary across different experiments due to temperature drift. The offset is removed from the raw measurements separately, and the calibration problem is reduced to:

(11) $$\underset{C}{\text{arg min.}}\;\;\frac{1}{N}\sum_{i=1}^{N}{∥{\hat{f}}_{i}-C{\hat{r}}_{i}∥}^{2}.$$

where ${\hat{f}}_{i}$ and ${\hat{r}}_{i}$ are the reference 6D forces and raw measurements respectively with the offset removed.

#### 2.2.2. Regularization

In ex situ calibration matrix estimation methods, the input data $(r_{i},f_{i}),\;i=1\ldots N$ are obtained by applying a set of known masses in known locations with the sensor mounted on a workbench. Thus, this kind of ex situ calibration matrix will be referred as *Workbench* matrix. Assuming the calibration performed on the sensor was correct, the new calibration matrix must be similar to the *Workbench* matrix. To enforce this assumption, a regularization term to penalize the difference with respect to the *Workbench* matrix is introduced. The new calibration matrix is obtained through the following optimization problem:

(12) $$C^{*}=\underset{C\in R^{6\times 6}}{\text{arg min.}}\;\;\frac{1}{N}\sum_{i=1}^{N}{∥{\hat{f}}_{i}-C{\hat{r}}_{i}∥}^{2}+\lambda {∥C-C_{w}∥}^{2}$$

where $C_{w}\in R^{6\times \rho }$ is the *Workbench* matrix given by the sensor producer, λ is used to penalize the regularization term and *N* is the number of data points in the dataset. The regularization is added in order to try to keep the calibration matrix as close to the *Workbench* matrix, but keeping an improved performance after the sensor is mounted on the robot.

The form of the solution is obtain following these steps:

- Consider the Matrix form of the least squares
  (13) $${∥F^{⊤}-CR^{⊤}∥}^{2}+\lambda {∥C-C_{w}∥}^{2},$$
  where $F^{⊤}\in R^{6\times n}$ is the matrix with the reference 6D forces where each columns is ${\hat{f}}_{i}$, $R^{⊤}\in R^{\rho \times n}$ where each column is ${\hat{r}}_{i}$.
- Given $X\in R^{m\times n}$, $\mathrm{vec}\left(X\right)\in R^{nm}$ indicates the column vector attained by stacking the columns of the matrix *X*. Due to the definition of vec(·), it follows that
  (14) $$\mathrm{vec}\left(AXB\right)=\left(B^{⊤}⊗A\right)\mathrm{vec}\left(X\right),$$
  where ⊗ is the Kronecker product.
- If we consider that $CR^{⊤}=I_{6}CR^{⊤}$, where $I_{6}$ is a 6 by 6 identity matrix, then, using the Kronecker property mentioned in Equation (14), we can put Equation (13) in the column vectorized form:
  (15) $${∥\mathrm{vec}\left(F^{⊤}\right)-\left(R⊗I_{6}\right)\mathrm{vec}\left(C\right)∥}^{2}+\lambda {∥\mathrm{vec}\left(C\right)-\mathrm{vec}\left(C_{w}\right)∥}^{2}.$$
- The minimum of a quadratic form take place when the derivative is equal to 0. Using vector differentiation properties, the solution to Equation (15) can be written as
  (16) $$\mathrm{vec}\left(C^{*}\right)={\left(K_{R}^{⊤}K_{R}+\lambda I_{6*6}\right)}^{-1}\left(K_{R}^{⊤}\mathrm{vec}\left(F^{⊤}\right)+\lambda \mathrm{vec}\left(C_{w}\right)\right),$$
  where $K_{R}=\left(R⊗I_{6}\right)$. It is important to notice that the size of *I* multiplying λ should match the length of $\mathrm{vec}(C_{w})$ which is a∗ρ, where *a* is the number of axis (six-axis for these type of sensors) and ρ is the number of raw signals.

#### 2.2.3. Adding Linear Variables

When considering other phenomena as linear variables the final form of the problem can be expressed as:

(17) $$C^{*},C_{t}^{*}=\underset{C\in R^{6\times 6}}{\text{arg min.}}\;\;\frac{1}{N}\sum_{i=1}^{N}{∥{\hat{f}}_{i}-\left(C\hat{r_{i}}+C_{t}t\right)∥}^{2}+\lambda {∥C-C_{w}+C_{t}-C_{t_{w}}∥}^{2}.$$

where $C_{t}\in R^{6\times 1}$ are the added linear variables calibration coefficients and $t\in R^{n}$ are the added linear variable values. In this case, the problem is not only to estimate the calibration matrix *C* and the offset *o*, but also $C_{t}$ which accounts for the temperature changes in the sensor.

Given that $C{\hat{r}}_{i}+C_{t}t=\begin{array}{c} \left[C,C_{t}\right] \end{array}\begin{array}{c} \left[{\hat{r}}_{i}t\right] \end{array}$ adding a linear variable can be seen as adding an extra raw signal to the previous described solution. What needs to be done is:

- Augment the raw measurements matrix *R* with the added linear value $R_{a}=\left[R,t\right],t\in R^{n}$, in *R* each column has all the raw measurements of a given raw signal.
- Augment the *Workbench* matrix by adding the coefficients regarding the added linear variable $C_{w_{a}}=\left[C_{w},C_{t_{w}}\right]$, where $C_{t_{w}}$ is the added linear variable value at the time of calibration. If is not available, is recommended to set it as a vector of zeros $0_{6\times 1}$.
- Since $C_{w_{a}}\in R^{6\times \rho +1}$ this should be reflected in $L=\lambda *I_{6*(\rho +1)}$, if the *Workbench* coefficients of the added linear variable $C_{t_{w}}$ are not provided, it is suitable to set the last *a* values in the diagonal(L) to 0. This avoids influencing the coefficients of the added variable with any previous information.
- The final form of the solution is
  (18) $$\mathrm{vec}\left({\begin{array}{c} C,C_{t} \end{array}}^{*}\right)={\left(K_{R_{a}}^{⊤}K_{R_{a}}+L\right)}^{-1}\left(K_{R_{a}}^{⊤}\mathrm{vec}\left(F^{⊤}\right)+L\mathrm{vec}\left(C_{w_{a}}\right)\right)$$

This formulation allows to easily expand the solution to *m* number of extra linear variables. The extra linear variable can have its offset removed or not. Assumptions can be made by taking the first value and consider it as the offset of that variable.

#### 2.2.4. Offset Estimation

Two methods are proposed to remove the offset from the estimation problem.

1. *In Situ offset estimation* proposed in [10], but instead of accelerometers measurements the force torque reference values estimated with the model of the robot are used.
2. *Centralized offset removal* is obtained by removing the mean value from the raw measurements ($\mu_{r}$) and the reference values ($\mu_{f}$).

In both cases, we end up with a modified version of the raw data in which the effect of the offset is removed. With a little abuse of notation we have:

(19) $${\hat{r}}_{i}=\left\{\begin{array}{cc} r_{i}-o_{r} & \mathrm{in}\;\mathrm{situ}\;\mathrm{offset}\;\mathrm{estimation}\\ r_{i}-\mu_{r} & \mathrm{centralized}\;\mathrm{offset}\;\mathrm{removal} \end{array}\right.$$

(20) $${\hat{f}}_{i}=\left\{\begin{array}{cc} f_{i} & \mathrm{in}\;\mathrm{situ}\;\mathrm{offset}\;\mathrm{estimation}\\ f_{i}-\mu_{f} & \mathrm{centralized}\;\mathrm{offset}\;\mathrm{removal} \end{array}\right.$$

where ${\hat{r}}_{i}$ and ${\hat{f}}_{i}$ are the data used to solve the model-based in situ calibration problem (12).

Each offset estimation type is based on a different assumption:

1. physical assumption: mass generates a sphere in the force space when making spherical movements.
2. mathematical assumption: taking out the mean from the dataset implies no offset in the calibration data.

### 2.3. Calibration Data Set

Two types of datasets that were used previously to calibrate a sensor.

- **Grid**: moving the legs creating a grid pattern while being on a fixed pole. The contact happens at the waist of the robot. The leg is not bent to avoid changes in the center of mass of the leg during the experiment.
- **Balancing Support leg**: doing an extended one foot balancing demo with widespread leg movements. The contact is on the support leg foot. Either right (BSR) or left (BSL) depending on the support leg.

A calibration dataset could be formed by one of these kinds of dataset or a combination of them.

### 2.4. Previous Results

Previous results showed that the sphere estimation type using the Grid dataset gave the best results when no temperature was taken into account [11]. Adding the temperature required considering more than one dataset to incorporate multiple temperatures. It was proven that adding the temperature led to a further increase in performance. Mixing types of datasets gave better results [14]. The in situ offset estimation type with temperature was shown to be better, followed closely by the *centralized offset removal* with or without temperature, using the same λ value.

In those tests, no temperature offset was considered. The validation datasets were collected the same day in between the calibration datasets. The results shown were about the external force estimation, but how this affects robot performance was not presented.

### 2.5. Experimental Platform

The experimental platform is the floating base robot iCub. It is a child-sized humanoid robot originally developed during the RobotCub European Project for research in embodied cognition [36] by the *iCub Facility* at the *Italian Institute of Technology*.

It has 53 degrees of freedom (DoF), weighs around 33 kg and is 104 cm tall. The DoFs are distributed as in the following way: six for each leg, three for the torso, six for the head and eyes, seven for each arm and nine for each hand. One additional servo motor is used to open and close the eyelids. For the calibration, only a subset of 32 DOFs (legs, torso, arms, and neck) are used. The version of iCub used is known as 2.5. It has Brushless Direct Current electric motor (BLDC) with an Harmonic Drive transmission, making them suitable for joint torque control.

**Figure 1:**
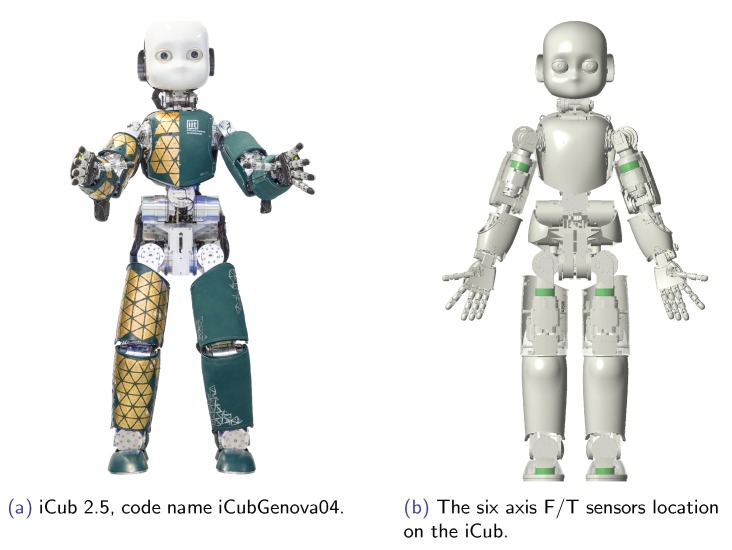
The floating base robot iCub. (**a**) the real robot can be appreciated showing the underneath skin techonology. (**b**) Shows the location of the FT sensors in the robot.

More details on the actuation and mechanics of the iCub 2.5 can be found in [4]. An image of the robot is presented in Figure 1.

The iCub has various sensors including inertial measurement units (IMU), force-torque (FT) sensors, cameras, microphones, joint encoders and tactile sensor arrays, that cover the surface of the robot. Six custom-made six-axis FT sensors [37]) are placed as shown in Figure 1. Only the force-torque sensors mounted on the legs and feet have temperature sensors. These sensors use silicon strain gauge technology.

The interface to interact with the iCub is through Yet Another Robot Platform (YARP). More specifically, YARP supports building a robot control system as a collection of programs communicating in a peer-to-peer way, with an extensible family of connection types (tcp, udp, multicast, local, MPI, mjpg-over-http, XML/RPC, tcpros, ...) that can be swapped in and out.

### 2.6. Force-Torque Sensing in the ICub

The force-torque sensing has five main uses in the iCub:

- To estimate the FT sensors offset before experiments.
- As a threshold to know if a stable contact has been established between the robot and the ground.
- To give the feedback to the low-level joint torque controller.
- To estimate dynamical quantities used by high-level controllers, such as the center of pressure (CoP) and the zero moment point (ZMP).
- To give feedback to high-level controllers.

*To Estimate the FT Sensors Offset before Experiments*

A typical sequence before using the robot, involves removing the offset when only one contact with the environment exists. This offset estimation requires the information from the mass vector. This can be imposed in a known robot configuration or measured using an IMU. This offset is then subtracted from the measurements. There are three possibilities to estimate the offset:

- The robot is hanging from the torso and mass is measured with IMU.
- The robot is standing on one leg and the mass is measured using IMU.
- The robot is standing on one leg and the mass is imposed to be acting in the axis normal to the ground.

*As a Threshold*

The simplest use is as a threshold. When the load on the sensors reaches a chosen value, the controllers change state assuming a stable contact has been achieved. The chosen value can be a percentage of the total body weight of the robot. Example of applications are balancing [38] and standing up [39].

*To Calculate Dynamic Quantities*

The forces acting on a moving robot can be separated into two categories: forces exerted by contact and forces transmitted without contact (mass and, by extension, inertia forces). The CoP is linked to the former, and the ZMP to the latter. Nonetheless, it has been shown that both points coincide [40]. Therefore, is possible to use the contact force information to calculate dynamic quantities such as the ZMP and by extension affect the estimation of the center of mass (CoM).

The CoP is defined as the point where the resultant force can be exerted with a zero resultant moment. When the contact is with a flat ground the CoP and ZMP, can be calculated as:

(21) $$P_{CoP}={}_{s}\tau /{}_{s}f,$$

where $P_{CoP}=P_{ZMP}$ is the CoP (ZMP), ${}_{s}\tau$ is the torque measurement of the FT sensor at the ankle and ${}_{s}f$ is the vertical force measurement of the FT sensor at the ankle.

Using the linear inverted pendulum model constraining the height of the CoM to be constant ($P_{CoG_{z}}$), the CoM dynamics can be estimated from the ZMP with the following equation:

(22) $${\ddot{P}}_{CoM}=\left(P_{CoM}-P_{ZMP}\right)\frac{g}{P_{CoM_{z}}},$$

The FT sensor measurements have a direct impact on the estimation of the ZMP and as a consequence in estimation of the CoM. This information is used in a walking controller [41].

*As Feedback for Joint Torque Controller*

**Figure 2:**
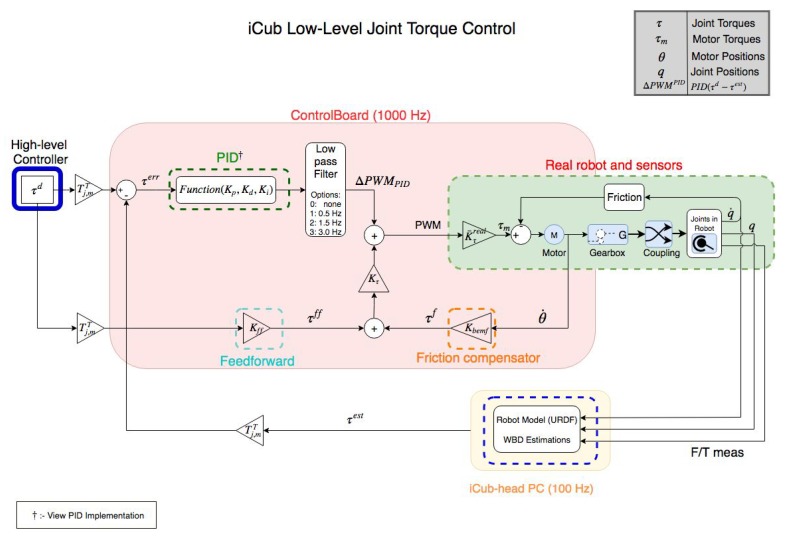
iCub’s Joint torque controller.

The scheme of this controller can be seen in Figure 2. Description of the variables in the scheme can be found in Table 1. It is a PID controller with friction compensation. The feedback values are the estimated joint torques using the measurements from the FT sensor [35].

*As Feedback for Jerk Controller*

A recent method for exploiting force-torque sensing is to use the estimated contact force as feedback to a high-level jerk controller of floating base systems with contact-stable parametrised force feedback [42].

The momentum rate-of-change equals the summation of all the external wrenches acting on the robot. In a multi-contact scenario, the external wrenches reduce to the contact forces plus the mass force:

(23) $$\begin{array}{c} \dot{H}=\sum_{k=1}^{n_{c}}A_{k}f^{k}-mge_{3}=Af-mge_{3},\\ A:=\;\left[A_{1},\ldots \;,A_{n_{c}}\right]\in R^{6\times 6n_{c}},\\ A_{k}=\left[\begin{array}{cc} I_{3} & 0_{3}\\ S({\;}^{I}o_{C_{k}}-{\;}^{I}o_{CoM}) & I_{3} \end{array}\right], \end{array}$$

where $H\in R^{6}$ is the robot’s momentum, $A_{k}\in R^{6\times 6}$ is the matrix mapping the *k*-th contact wrench to the momentum dynamics, ${}^{I}o_{C_{k}}\in R^{3}$ is the origin of the frame associated with the *k*-th contact, and ${}^{I}o_{CoM}\in R^{3}$ is the CoM position.

By considering the invertible parametrization $f^{k}=\phi \left(\xi^{k}\right)$, it is possible to ensure the friction cone constraints while avoiding to use inequality constraints. The mentioned controller uses the following optimisation problem:

(23a) $$\begin{array}{l} \underset{u=\dot{\xi }}{\text{arg min.}}\;{∥{\ddot{H}}^{d}-\ddot{H}\left(\dot{\xi }\right)∥}^{2}\\ \mathrm{subject}\;\mathrm{to}: \end{array}$$

(23b) $$\begin{array}{c} \ddot{H}\left(\dot{\xi }\right)=\dot{X}\phi \left(\xi \right)+X\delta_{\xi }\phi \left(\dot{\xi }\right), \end{array}$$

(23c) $$\begin{array}{c} {\ddot{H}}^{*}={\ddot{H}}_{d}-K_{d}\left(\dot{H}-\dot{H_{d}}\right)-K_{p}\left(H-H_{d}\right)-K_{i}\int_{0}^{t}\left(H-H_{d}\right)dt. \end{array}$$

where $K_{d},K_{p},K_{i}\in R^{6\times 6}$ are symmetric and positive definite matrices, $H_{d}$ is the reference momentum, *X* is the adjoint transformation matrix from the contact to the base of the robot [43] and *u* is the control input.

The contact forces are calculated using the FT measurement. In this controller, the FT sensor measurements are directly used as feedback since they affect directly the computation of the momentum rate-of-change.

## 3. How to Better Exploit the Model Based In Situ Calibration Method

One of the aims of the paper is to provide useful tips to use the calibration method more effectively. The effectiveness of this tips is shown using real robot experiments.

### 3.1. New Offset Estimation

From the resulting generic formulation to add linear variables, described in Section 2.2.3, it is possible to formulate the problem in a way that the offset does not have to be removed from the calibration data before computing the least squares solution. This offset estimation type is named *one shot estimation*. It estimates the offset as another set of coefficients of the calibration matrix by adding a linear variable in which the reference values are all 1. Contrary to the other two offset estimation types it makes no assumptions and allows the least squares to simultaneously solve the offset with the calibration matrix.

### 3.2. Understanding Sensor Excitation

Based on availability and excitation of the sensor, two more types of datasets were studied.

- **Balancing Non-Support leg**: doing an extended one foot balancing demo with widespread leg movements. The contact is on the other leg foot. Either left (BNSL) or right (BNSR) depending on the support leg.
- **Z-Torque**: doing movements designed to generate torques around the z axis, while the robot is on a fixed pole.

**Figure 3:**
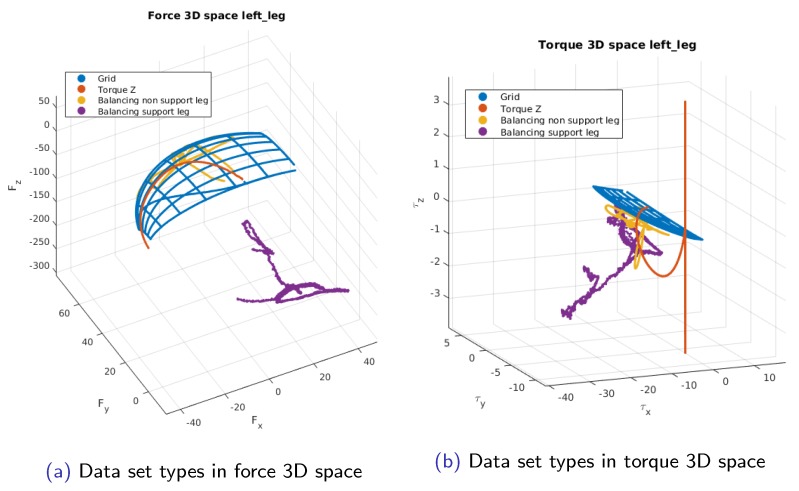
Images of the all the dataset types considered for calibration in their respective 3D space.

After adding this two dataset types the resulting 3D force space and 3D torque space with all types of datasets are depicted in Figure 3. Studying the results of these new types of datasets allows to understand how the calibration results are affected by the excitation of the sensor represented in the 3D force space and 3D torque space.

### 3.3. The Constant Offset Hypothesis

Considering that most of the drift is assumed to be caused by temperature, it follows that by properly compensating the temperature drift, the offset of the sensor itself should be time invariant. This can be proven by using the offset estimated at the time of the calibration and applying it on some other time while the sensor is subjected to different temperatures.

### 3.4. Exploiting Model-Based In Situ Method in a Robot

The result from the Model-based in situ calibration is a new calibration matrix. It is when using this calibration matrix that the improved measurements are obtained. Therefore, the new calibration matrix should be used somehow by the robot to obtain the improved measurements and better dynamic performance as a consequence.

#### 3.4.1. Secondary Calibration Matrix

It is possible that is not easy to change the current calibration matrix of the sensor. In these cases, the proposed solution takes the form of a secondary calibration matrix. The secondary calibration matrix is the required transformation of the current calibration matrix to the new calibration matrix. It requires the knowledge of the current calibration matrix used by the sensor. It is calculated as follows:

(24) $$C=C_{s}*C_{w}\to C_{s}=C*C_{w}^{-1}$$

Before using the values obtained through force-torque sensing, they can be corrected by pre-multiplying with the secondary calibration matrix. This way the measurements used by the robot are the same as if the sensor was calibrated using the in situ calibration matrix.

#### 3.4.2. Adding the Temperature and Offset

The contribution of the temperature can be added separately. In case no temperature calibration is available, these coefficients are loaded as zeros by default. This is helpful in cases where temperature measurements are available for some but not all sensors.

Instead of estimating the offset before the experiment, the offset estimated by the calibration method can be used by summing the offset after the new calibration matrix and temperature compensation is applied.

## 4. Methodology

The improvement in the measurements among the different estimation types is compared among the different kinds of possible calibration datasets to select the best way to improve the FT sensor performance. For comparison, results using the *Workbench* matrix are included as an estimation type in its own. At a first stage, the different dataset types were compared on their own. To further test the robustness of the in situ estimation, a different set of calibration and validation datasets were collected.

### 4.1. Data Sets Used

The validation datasets were taken on two different days, both different from the day the calibration datasets were collected. This was done to test the robustness to possible different ambient conditions. The datasets and their temperatures are showen in Table 2.

The calibration datasets were grouped into:

- noTz, as indicated by name none of the Z-torque datasets were included.
- onlySupportLegs, from the balancing datasets, only the support leg was included. All other dataset types were included.
- AllGeneral, all dataset types were included.

The reasoning behind this arrangement of datasets is to see what combination of datasets provides the best results. Since it was proven before that Grid and Balancing together improve the calibration, the variables to test are the inclusion of Z-torque and non-support leg datasets.

### 4.2. Defining Estimation Types

Each strategy of offset estimation is considered an estimation type. Including temperature as a linear variable (wT) or not (nT) in the estimation are also considered different estimation types. If the temperature is considered, it is possible to take the first value as an offset (rTO) or not (dTO). Considering the three offset removal possibilities, adding the temperature as a linear variable to each of them and the temperature offset option results in the following nine estimation types:

- Sphere with no temperature (**SnTdTO**): Refers to the fact that the in situ offset removal is obtained by expecting a sphere in the force space when generating circular motions. No temperature considered.
- Centralized with no temperature (**CnTdTO**): Refers to the centralized offset removal method without considering temperature.
- One Shot with no temperature (**OnTdTO**): Refers to estimate the offset and the calibration matrix at the same time without considering temperature.
- Sphere with temperature (**SwTdTO**): Refers to including temperature into the sphere type. But no temperature offset is considered.
- Centralized with temperature (**CwTdTO**): Refers to including the temperature into the centralized type. But no temperature offset is considered.
- One Shot with no temperature (**OwTdTO**): Refers to estimate the offset and the calibration matrix at the same time considering temperature. But no temperature offset is considered.
- Sphere with temperature (**SwTrTO**): Refers to including temperature into the sphere type. Removing temperature offset.
- Centralized with temperature (**CwTrTO**): Refers to including the temperature into the centralized type. Removing temperature offset.
- One Shot with no temperature (**OwTrTO**): Refers to estimate the offset and the calibration matrix at the same time considering temperature. Removing temperature offset.

**Figure 4:**
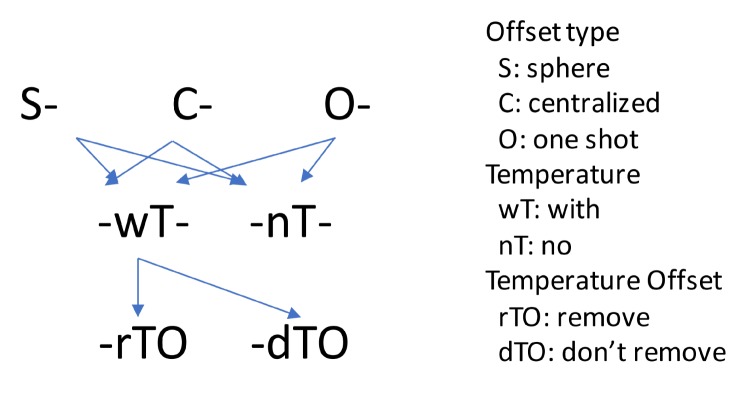
Estimation types name logic.

The logic behind the estimation type names can be seen in Figure 4.

### 4.3. Evaluation Description

The evaluation could be roughly divided in three parts: one to observe the results of each estimation type, another to check the expected improvement on the robot of the generated calibration matrices and a third to verify the impact of using the contributions in a real robot. The sensor to calibrate is located near the hip of the left leg of an iCub robot.

#### 4.3.1. Estimation Type Validation

To understand better the behavior of the estimation types three comparisons are done. The first uses the mean square error (MSE) calculated between the force-torque data using the new calibration matrix and the model-based estimated data. A lower value would indicate a better fitting of the data. Mean Square Error (MSE) of each axis is calculated as follows:

(25) $$MSE=\frac{1}{N}\sum_{i=1}^{N}{\left(f_{i}^{r}-{\hat{f}}_{i}^{c}\right)}^{2},$$

where $f_{i}^{r}$ is the 6D force reference vector and ${\hat{f}}_{i}^{c}$ is the 6D force vector obtained using the estimated calibration matrix of each estimation type. A second way is to compute the mean of the absolute value of the difference between matrices. This is to get a general idea of how much the calibration matrices differ one from the other. The third way is looking at the offset values. This is to see how the different estimation types affect the estimation of the offset. Although there is no ground truth for the offset to serve as a comparison, similarity in the offset values might indicate a general idea of what the true offset might be.

#### 4.3.2. External/Contact Force Estimation Validation

The selection of the best calibration matrix is done using the contact force validation described in a previous work [11]. It emulates the contact force estimation algorithm in an offline environment. This form of evaluation permits to check the performance of the mean value of magnitude of the force or the value of each axis over a set of validation experiments. This is relevant since there is no guarantee that a λ value or the same type of estimation gives the best results in all axes. The reason is that each axis can be seen as a separate problem.

The λ values used are: [0, 1, 5, 10, 50, 100, 1000, 5000, 10,000, 50,000, 100,000, 500,000, 1,000,000]. The λ values where selected to span a reasonable range based on the tests to make *C* converge to $C_{w}$, which happened when λ≈1e+08. The validation is performed on each combination of calibration dataset (3), estimation type (9), and λ value (13). In total 352 calibration matrices are evaluated counting the *Workbench* matrix.

#### 4.3.3. Impact on Robot Behavior Evaluation

In most cases the dynamic behaviors of iCub are obtained through high-level controllers. They contain many tuning parameters that affect the behavior of the robot. The measurements of the sensor are used mainly in an indirect way by the high-level controllers. Therefore, finding quantitative measures of the improvement in the dynamic motions of the iCub caused by the in situ calibrated sensor is a challenging. Nonetheless, from the uses of the FT sensor on iCub, three quantitative methods were used:

- Contact force values when switching contact.
- Accepted gains values used in the low-level controller.
- Simultaneous online comparison of force torque measurements performance using different offsets.

*Contact force values when switching contact*

The test performed consist in switching from single support to double support.

If the offset is calculated with the robot standing on one foot the sequence is:

- Single support → double support → other single support.

Instead if the offset is calculated with the robot in the air the sequence is:

- Double support → single support → double support → other single support.

Since the robot is standing on flat ground it is expected that the only force acting on the robot feet is mass on the z axis. With no other force acting on the robot the forces in x and y should cancel each other in double support or be 0 when on single support. With this as a ground truth, is possible to evaluate the estimated contact forces at the feet when switching.

*Accepted gains values used in the low-level controller*

The robot was tuned to the maximum gain value in which the robot is able to perform the balancing demo in a satisfactory way. Beyond a certain value, the robot is observed to vibrate and fall. For this test, the high level gains of the controller are kept the same and only the low-level controller gains are changed. A higher gain value indicates that the robot is able to rely more on the measurements of the sensor as feedback. Thus, being able to use higher gains is better if it does not introduce unstable behaviors.

*Simultaneous online comparison of force torque measurements performance using different offsets*

For this experiment the offsets used were estimated with a month difference with respect to the experiment date. The comparison between the expected FT value and the actual value obtained after applying both the offset and the secondary calibration matrix correction. Three different offsets where used:

- offset${}_{noTemp}$: is the estimated offset from the best calibration matrix without temperature one month before experiments.
- offset${}_{temp}$: is the estimated offset from the best calibration matrix using temperature one month before experiments.
- online: is the offset calculated on the online on the robot the day of the experiment when the robot was just turned on. The temperature was 26 ${}^{\circ }C$. It uses the same secondary calibration matrix as the one from offset${}_{noTemp}$.

Three simultaneous estimation applications are launched. One for each type of offset used. The ground truth is calculated offline in the sections of the experiments in which there was only one contact with the environment. Multiple experiments were performed on the robot in the span of three hours to allow the FT sensors to heat up.

## 5. Results and Discussion

### 5.1. Estimation Types Behavior

To verify the behavior of the estimation types only the results from a single calibration dataset is showed. The one selected is the onlySupportLegs dataset. Nonetheless, the results extend to the other two calibration datasets.

#### 5.1.1. MSE Error

The MSE error of each estimation type is shown in Table 3. This value is linked to the calibration dataset in which the calibration matrix was estimated and is affected by the number of calibration points. Although the value can not be compared among datasets, the tendencies are similar. One of them is how the MSE drops by taking temperature into account. For sphere types (SnTdTO, SwTdTO and SwTrTO), removing the temperature offset further reduces the error while for the centralized (CnTdTO, CwTdTO and CwTrTO) and OneShot (OnTdTO, OwTdTO and OwTrTO) types there seems to be no benefit. It can be observed that the fitting from the centralized and OneShot types are identical. For the calibration datasets considered, the Centralized/OneShot types give better results in general. The only exceptions appear in the noTz calibration dataset. In this dataset the sphere types have an slight advantage in three axis: $f_{x}$, $\tau_{x}$ and $\tau_{z}$.

#### 5.1.2. Calibration Matrix Differences

Table 4, shows a comparison between the different estimation types including the *Workbench* matrix. In general, the highest values are obtained when comparing with the *Workbench* matrix. From these, the most different matrices are the ones obtained not considering temperature. Sphere type with no temperature (SnTdTO) is the most different of all with respect to the *Workbench* matrix.

Is possible to see that the resulting calibration matrix from centralized types is the same as the one obtained through One shot types. The calibration matrix does not change between using the temperature offset (CwTrTO) or not (CwTdTO) for the centralized types.

**Figure 5:**
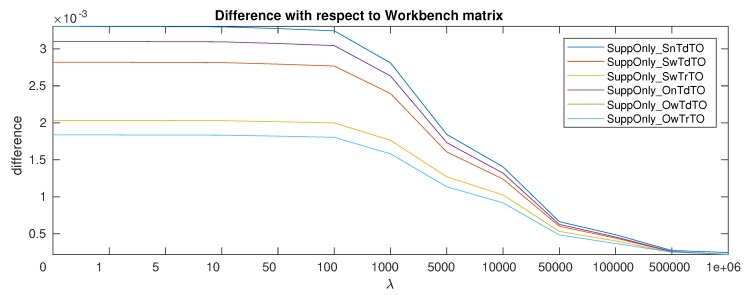
Difference between estimation types and *Workbench* matrix while increasing λ.

Taking into account the λ values and looking at the difference with respect to the *Workbench* matrix as shown in Figure 5. The effect of the regularization parameter becomes clear. The higher the value the smaller the difference with respect to the *Workbench* matrix.

Is worth noticing that taking into account the temperature makes the matrix more similar to the *Workbench* matrix even for λ=0. Since the new calibration matrix is expected to be relatively close to the *Workbench* matrix, this similarity even when no penalization is used can be interpreted as a sign of better calibration. Considering this it can be seen that the sphere estimation types benefits from adding the temperature and even more from taking out the temperature offset. The centralized types benefit from adding the temperature, even if there seems to be no added benefit from considering the temperature offset.

#### 5.1.3. Offset Comparisons

The estimated offsets can be seen in Table 5. It shows that taking into account the temperature offset changes the results of the offset estimation. The estimated offsets without temperatures are not very different between them. Something similar can be seen for the offset obtained considering the temperature offset. In contrast, the offset including temperature, but neglecting the temperature offset, has considerably different behavior between the sphere and the other types.

The estimate offset varies more on the Centralized and Oneshot types. Is noteworthy that the fitting of the data is equal even if the offsets are different as seen from the MSE error in Table 3. The temperature coefficients and the calibration matrix are the same. What changes is the contribution from temperature. It seems that the offset estimation of the Centralized/OneShot types collects both the force-torque offset and the temperature offset into the force-torque offset if no temperature offset is explicitly removed. Therefore using the temperature offset might facilitate to gauge the offset purely in terms of forces with not temperature influence.

### 5.2. Contact Force Validation

This validation was performed twice. One using only the calibration matrices estimating the offset in a few of the samples and the other using also the estimated offsets. The results of the contact force validation are shown in Table A1 and Table A2. From the evaluation of the estimation types behavior is clear that the Centralized types and the Oneshot types give the same result. Because of this only the Sphere types and the OneShot were considered.

#### 5.2.1. Using Only Estimated Calibration Matrices

In this case, the offset is calculated taking some samples of the test experiments in which is known the robot is on one foot and not moving or moving slowly. The offset calculation includes not only the forces but also the temperature if coefficients are available.

**Figure 6:**
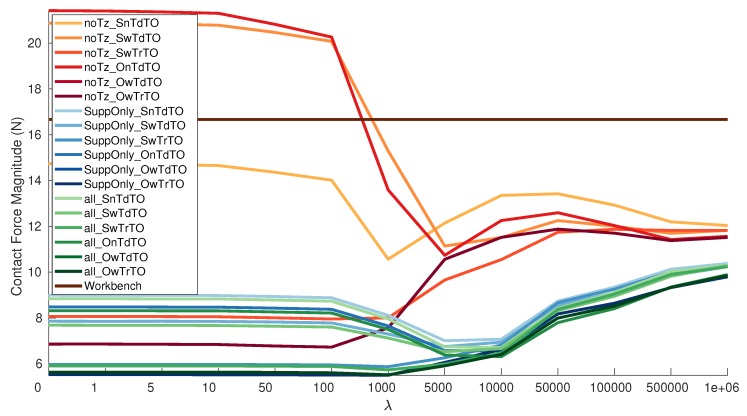
Magnitude contact force versus λ using estimated only Calibration Matrices, Table A1.

The best result is achieved by OwTdTO and OwTrTO with 5.498 N with λ=1000 as the average magnitude of the contact force. In general, better results are achieved by including the temperature and using the OneShot estimation types. The results with the SwTrTO are also close to the best result. The SwTdTO type in noTz dataset has the worst performance. The error is reduced greatly by removing the temperature offset, as seen from the fact that SwTrTO has a consistently lower value than SwTdTO in each dataset. Therefore removing the temperature offset is relevant for the sphere types. The added benefit of the previous calibration matrix information seems more relevant for estimation types that do not consider the temperature. It is also possible to appreciate that increasing λ is beneficial up to a certain point after which it increases the error. This is expected since the *Workbench* is considered to be correct when the sensor was unmounted, so becoming similar to it has benefits. On the other hand, the mounting changed the effectiveness of it, so being to close has lower performance. This is quite clear in Figure 6. The contact force magnitude of the new calibration matrices is a considerable lower than the *Workbench* for all the validation datasets.

#### 5.2.2. Using Also Estimated Offsets

**Figure 7:**
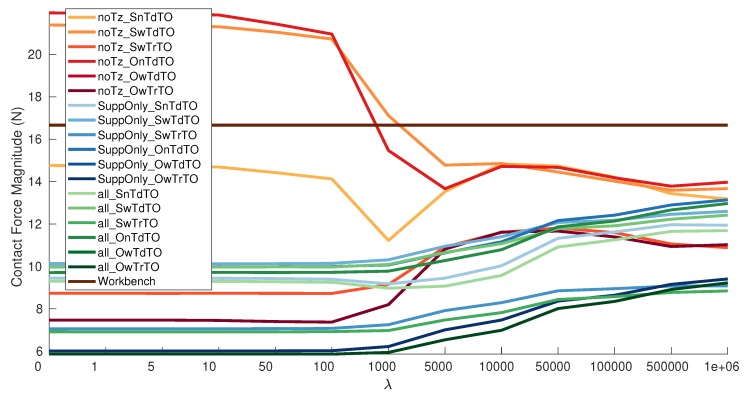
Magnitude contact force versus λ using also estimated Offset, Table A2.

When using the estimated offset, the best result is obtained by OwTrTO with λ 100 on the calibration dataset *AllGeneral*, Table A2. A graphic representation of the results can be seen in Figure 7. The average magnitude of the external force is 5.8647 N which is close to the one obtained estimating the offset on the validation dataset. It hints that the estimated offset can be replace the offset calculated before the experiment.

#### 5.2.3. Analysing Dataset Types

Grouping the results by dataset, the calibration datasets can be ordered from best to worst in the following order: AllGeneral, onlySupportLegs and noTz. The behavior of a group of results by dataset is clear using the pallets of colors in Figure 6 and Figure 7. There is a big improvement when adding the Z-Torque dataset and just a small improvement from adding the Balancing Non-Support Leg on top of that. Showing that the Z-Torque gives relevant new information to the calibration dataset, while the Balancing Non-Support Leg adds few more information. Therefore, for the considered dataset types the optimal combination is composed of Grid, Z-Torque, Balancing Support Leg. The Balancing Non-Support Leg can be considered optional and not strictly required. The fact that the best results not using the estimated offset is without using the Balancing Non-Support Leg dataset reinforces the previous statement. It can be seen that adding the temperature creates a clear difference in the results of a dataset. It almost divides each different calibration set results in two.

These results are congruent with the space each dataset covers in the forces and torques 3D space. In Figure 3, is possible to see that Balancing Non-Support leg is more or less contained between the Grid and Balancing Support Leg. The other three dataset types are clearly different among them. Therefore is possible to use that graphical representation to gauge the expected usefulness of a dataset.

#### 5.2.4. Results by Axis

Table 6 shows the best results by axis and the performance of the *Workbench* matrix. From the difference in the results with respect to the *Workbench* is possible to see that the most affected axis by the mounting are $f_{x}$ and $f_{y}$. Is possible to see that $f_{z}$, $\tau_{x}$ and $\tau_{y}$, actually perform better using the estimated offset. The fact that only the $f_{y}$ and $f_{z}$ get better results in both cases taking into account the temperature might imply these are the axis mainly affected by the temperature drift.

**Figure 8:**
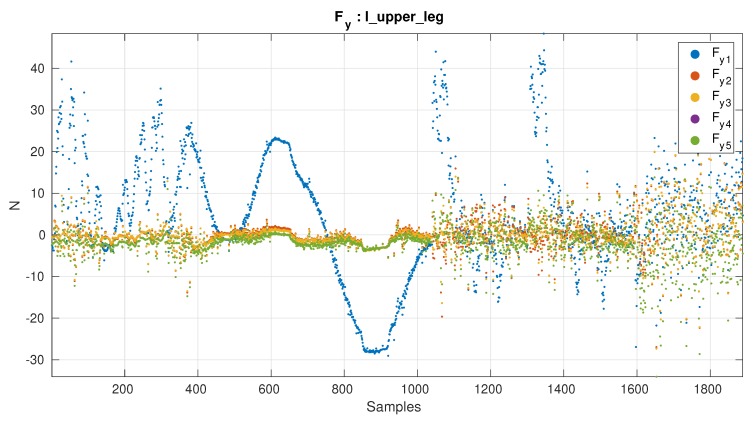
Force y-axis. Axis with biggest improvement. Calibration matrices: (1) Workbench matrix, (2) Best not using estimated offset, (3) Best by Axis not using estimated offset, (4) Best using estimated offset, (5) Best by Axis using estimated offset.

**Figure 9:**
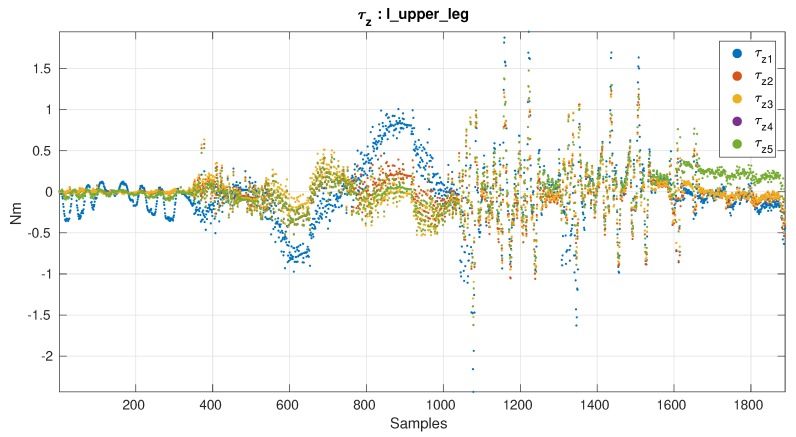
Torque z-axis. Axis with the least improvement. Calibration matrices: (1) Workbench matrix, (2) Best not using estimated offset, (3) Best by Axis not using estimated offset, (4) Best using estimated offset, (5) Best by Axis using estimated offset.

Figure 8 and Figure 9 show the axes that improved the most and the least respectively by the calibration procedure. Although the variation in lambda value is big, looking at Figure 8 and Figure 9 it can be seen that the difference among the best solutions is small.

### 5.3. Observed Improvements in Floating Based Robots

By improving the measurements of the six-axis FT sensor through in situ calibration, it was possible to see improvements in the behavior of the floating base robot iCub.

#### 5.3.1. Contact Force Coherence When Switching Contact

Results are shown in Table 7 and Table 8. It can be observed that using the in situ calibrated sensors, reduces the error in the contact forces and is more coherent when switching from a contact to another. This behavior can also be observed in the attached video. The red lines are the estimated values for the external force. There is a smoother transition in the length of the lines during the switching of double support and single support when using the new calibration matrix. Also the contact forces remain close to the expected values during the execution of the demo. This observed by how small the red lines are on the parts of the robot not in contact.

#### 5.3.2. Increase in the Accepted Gains of the Low-Level Controller

The balancing demo [38] was observed to have oscillations of the robot when reaching the different pre-defined position tasks. After the six-axis FT measurements were improved, it was feasible to increase the gains of the low-level controller between 10 and 15 units depending on the joint. In some joints, this signified a 50% increase. It was observed that the movements of the robot seemed more defined and there were clearly fewer oscillations and less time required to switch to the next position task. Moreover, it was clear that the same gains without the in situ estimated matrix make the robot fail immediately. Thanks to the reliability of the measurements, the feedback of the low-level controller is more useful to control the robot creating a faster convergence to the desired value. This can be observed in the attached Video S1.

#### 5.3.3. Simultaneous Online Comparison of Force Torque Measurements Performance Using Different Offsets

**Figure 10:**
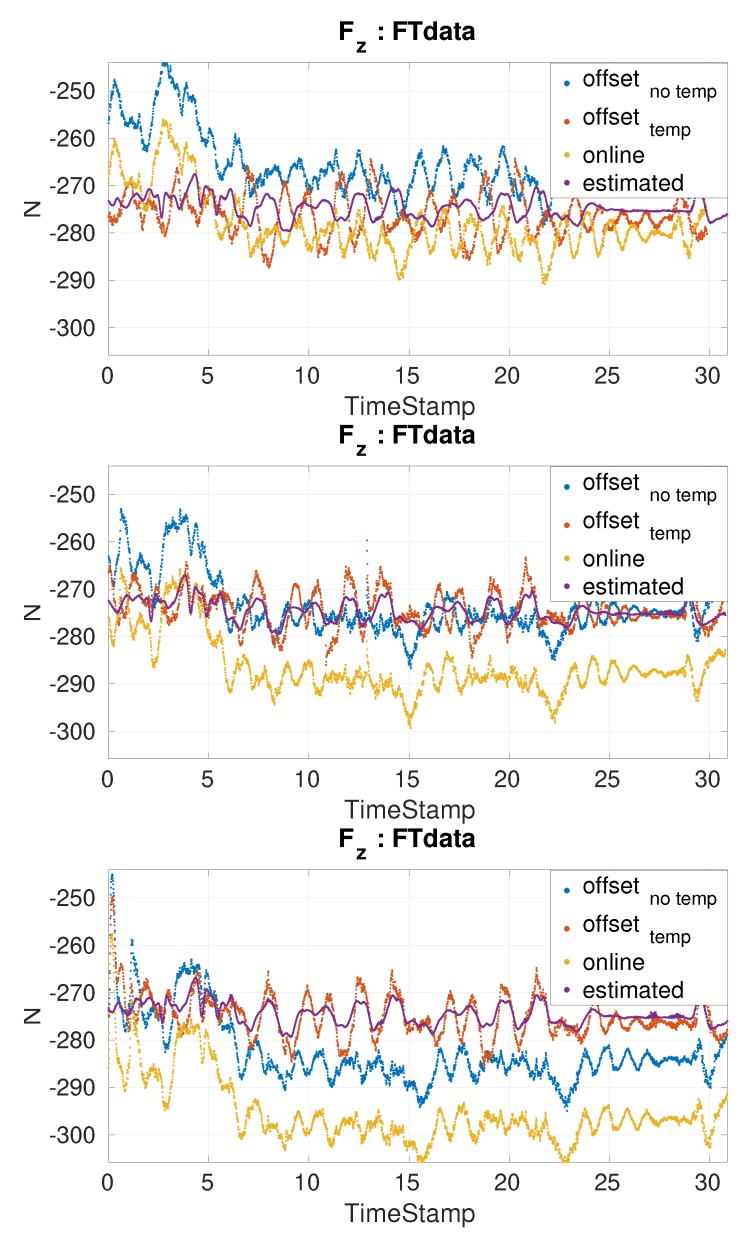
Forces on the z axis for balancing experiments at different temperatures. From left to right the respective temperatures were: 26.6 ${}^{\circ }C$, 36.2 ${}^{\circ }C$ and 43.5 ${}^{\circ }C$.

Based on the resulting coefficients of the temperature, $C_{t}$ = [$f_{x}$−0.0933, $f_{y}$ 0.2048, $f_{z}$ 1.3342, $\tau_{x}$ −0.0155, $\tau_{y}$ 0.0027, $\tau_{z}$ 0.0039], it can be seen that the most affected axis is the force on z. The comparison for $f_{z}$ axis is shown in Figure 10. The online offset is close to the estimated value only at 26.6 ${}^{\circ }C$. This temperature is similar to the temperature when the offset was estimated. The average temperature at the sensors after a a few mins is around 36.2 ${}^{\circ }C$. At this temperature, the offset estimated without temperature has very similar results as the one with temperature. When the robot reaches 43.5 ${}^{\circ }C$ only the offset computed with temperature is still close to the estimated value as it has been for all the temperatures. Therefore, the need to re-estimate the offset before every experiment disappears.

#### 5.3.4. Allow the Use of FT Measurements as High Level Controller Feedback

Besides the quantitative evaluations of performance, an important qualitative behavior was observed when the measurements were used in high level controllers. During the experiments for the jerk control of floating base systems with contact-stable parametrised force feedback, it was observed that without the secondary calibration matrices the error in the estimated external forces was so big that the controller was unable to perform the experiments. Using the secondary calibration matrices reduced the error to ±2.5N [42]) in the worst case. The error was low enough to successfully perform the experiments by using the regularization term.

### 5.4. Comparison with Other Methods

Compared to other in situ calibration methods [10], the Model based in situ calibration method avoids the need to install other sensors or specialized structures on the robot to perform the calibration. This avoids effort and possible human errors introduced in the estimation of the position of the other sensors with respect to the FT sensor.

In the previous Model Based formulation without temperature [11], less amount of data was used to calibrate the sensor. When considering only one type of dataset for calibration, the sphere types outperform the centralized ones. If more than one type of dataset was to be combined, it needed to be collected immediately one after the other for having the assumption of same offset still valid for both datasets. It was observed that even after the sensors were calibrated the offset estimated during calibration not suitable to use on the robot. The reason was that the measurements would drift and the estimated offset would not be valid after a short time. This was resolved by introducing the temperature as linear variable [14].

In the previous paper of model-based in situ calibrations with temperature compensation, results showed that the sphere types still had a certain advantage over centralized types. In those experiments, the amount of data used in calibration had less types of datasets equivalent to the noTz dataset here. By including the new types of datasets better results were obtained. Also, the one shot gave better performance than the sphere type. This shows a correlation between estimation type and types of calibration data used. The experiments described here show that datasets for calibration can be taken in different days in different conditions. This makes the data collection less restrictive despite the need for more data types. In the previous validation dataset, the offset was estimated at the beginning of the datasets. This is similar to the performance of the online estimated offset. This masked the difference in performance of the centralized offset with temperature and without. In here, the benefits of the method are further highlighted by showing the impact in the performance of floating base robots due to better FT measurements and the advantages of using the temperature are extended.

## 6. Conclusions

The developed algorithm has been proven to improve the measurements of the sensor and the dynamic behavior of a floating base robot. It successfully accounts for temperature drift and can be extended to account for other lineal phenomena, allowing controllers to reliably use the sensor measurements as feedback, be it directly or indirectly.

The new offset formulation is shown to give identical results as the centralized offset with the added benefit that the offset and calibration matrix are estimated simultaneously. The graphic representation of the sensor excitation in the 3D force and torque space has proven useful to provide intuitive insight into the comprehensive excitation of the sensor. In the Figure 3, it is evident to see that Z-Torque type of dataset provides new information. It is also visible that the Balancing Non-Support Leg gives redundant information. This was confirmed when looking at the results grouping by a calibration dataset. Considering it is possible to generate random movements of the robot and then evaluate if the dataset provides new useful data, this avoids the need to carefully design the joint trajectories for the calibration data. Since the approach is model-based, robot simulations can easily provide this graphic sensor excitation representation. The use of the secondary matrix allows a simple non-intrusive way to provide the improved measurements to the robot. It was shown that by adding the temperature we are able to use the offset of the sensors as a constant. This proves that the drift is mainly generated by the temperature. Furthermore, using offsets that were estimated a month in advance proves the robustness of the estimation of both calibration matrix and offset. It also demonstrates a higher reliability of the sensor measurements. The possibility to use a constant offset eliminates the need to estimate the offset before every experiment. This is especially useful for floating base robots that have a harder time anticipating the exact time of contact. It minimizes the preparation steps for using the robot and allows to do longer experiments without the need to stop. The improvement in robot performance is clear from the contact force coherence when switching or the fact that low and high-level controllers are able to perform better when using the FT measurements after in situ calibration.

The relationship between the regularization parameter and performance is clearly shown. This may allow to further refine and guide the selection of regularization parameters. The comparison with previous results suggests that the relevance and impact of each offset estimation strategy may be linked to the amount of data available. For small amounts of data, the physical assumption gives the highest improvements. With bigger amounts of data having no assumptions to estimate more accurately the calibration. The loss of effectiveness when using the physical assumption could be related to the fact that it does not consider temperature at all for offset estimation.

As future work, ways to account for nonlinearities will be explored. Studying the dynamical response of the sensor is also interesting and might provide further improvements in the performance of the sensor.

## Figures and Tables

**Table 1:** Variables used in the joint torque controller scheme (1: Duty Cycle Percentage.).

	Description	SI Unit
Gains		
$K_{p}$	Coefficient for the proportional term of PID	DC%^1^/N m
$K_{d}$	Coefficient for the derivative term of PID	DC%s/N m
ine $K_{i}$	Coefficient for the integral term of PID	DC%/$N\;m\;s^{-1}$
$K_{PWM}$	Transformation matrix between PWM and joint torque	N m/DC%
$K_{\tau }$	$K_{\tau }=1/K_{PWM}$	DC%/N m
$K_{bemf}$	Coefficient of Viscous friction	$N\;m\;s{\mathrm{rad}}^{-1}$
${\bar{K}}_{\tau }^{real}$	Transformation matrix between PWM and Motor torque	N m/DC%
Torque variables		
$\tau^{d}$	Joint Torque set as a reference via high-level controller	N m
$\tau^{est}$	Joint Torque estimated via WBD in the firmware	N m
$\tau^{PID}$	Joint torque error after passing through the PID	N m
$\tau^{ff}$	Feed forward term for the joint torque	N m
$\tau^{f}$	Joint torque term for compensating friction	N m
$\tau_{m}$	Motor torque obtained by transformation of PWM	N m
$\tau_{bias}$	Bias Force= CoriolisForce+GravityForce	N m
Other variables		
*q*	Joint Position	${}^{\circ }$ or rad
$\dot{q}$	Joint velocity	${}^{\circ }s^{-1}$ or $\mathrm{rad}s^{-1}$
θ	Motor shaft Position	${}^{\circ }$ or rad
$\dot{\theta }$	Motor shaft velocity ${}^{*}$	${}^{\circ }s^{-1}$ or $\mathrm{rad}s^{-1}$

**Table 2:** Used Data sets.

Type	Day	Temperature ${}^{\circ }$C	
		Start	End
Validation Data Sets			
Balancing Support Leg	1	38.0	38.2
Balancing No Support Leg	1	38.3	38.4
Grid	2	27.3	27.7
Z-Torque	2	27.7	27.8
Balancing Support Leg	2	34.7	34.8
Calibration Data Sets			
Grid	3	28.8	29.7
Grid_2	3	42.2	41.9
Z-Torque	3	29.7	29.8
Z-Torque_2	3	41.9	41.8
Balancing Support leg Left	3	30.8	31.0
Balancing Support leg Left_2	3	41.8	41.8
Balancing No Support leg Left	3	31.4	31.6
Balancing No Support leg Left_2	3	41.9	41.8

**Table 3:** Mean Square Error on same Calibration dataset.

EstimationType	$f_{x}$ (N)${}^{2}$	$f_{y}$ (N)${}^{2}$	$f_{z}$ (N)${}^{2}$	$\tau_{x}$ (N m)${}^{2}$	$\tau_{y}$ (N m)${}^{2}$	$\tau_{z}$ (N m)${}^{2}$
SnTdTO	12.1358	3.4528	62.1595	0.1222	0.0826	0.0299
SwTdTO	8.1290	3.4495	41.7705	0.1193	0.0781	0.0299
SwTrTO	10.8075	3.3826	5.7261	0.1192	0.0823	0.0299
CnTdTO	7.9941	3.4504	56.4681	0.1202	0.0764	0.0298
CwTdTO	8.0358	3.3441	3.5042	0.1188	0.0759	0.0297
CwTrTO	8.0358	3.3441	3.5042	0.1188	0.0759	0.0297
OnTdTO	7.9941	3.4504	56.4681	0.1202	0.0764	0.0298
OwTdTO	8.0358	3.3441	3.5042	0.1188	0.0759	0.0297
OwTrTO	8.0358	3.3441	3.5042	0.1188	0.0759	0.0297

**Table 4:** Mean Absolute difference among estimation types Mean Absolute difference (Values are at $10^{-4}$)  between estimation types, including *Workbench* matrix.

EstimationType	Workbench	SnTdTO	SwTdTO	SwTrTO	CnTdTO	CwTdTO	CwTrTO	OnTdTO	OwTdTO	OwTrTO
Workbench	0	33.0389	28.1952	20.3309	31.0110	18.3735	18.3735	31.0110	18.3735	18.3735
SnTdTO	33.0389	0	6.9890	15.9404	2.8109	16.4152	16.4152	2.8109	16.4152	16.4152
SwTdTO	28.1952	6.9890	0	10.2228	4.5113	10.7766	10.7766	4.5113	10.7766	10.7766
SwTrTO	20.3309	15.9404	10.2228	0	14.3387	2.1403	2.1403	14.3387	2.1403	2.1403
CnTdTO	31.0110	2.8109	4.5113	14.3387	0	14.5363	14.5363	0	14.5363	14.5363
CwTdTO	18.3735	16.4152	10.7766	2.1403	14.5363	0	0	14.5363	0	0
CwTrTO	18.3735	16.4152	10.7766	2.1403	14.5363	0	0	14.5363	0	0
OnTdTO	31.0110	2.8109	4.5113	14.3387	0	14.5363	14.5363	0	14.5363	14.5363
OwTdTO	18.3735	16.4152	10.7766	2.1403	14.5363	0	0	14.5363	0	0
OwTrTO	18.3735	16.4152	10.7766	2.1403	14.5363	0	0	14.5363	0	0

**Table 5:** Offsets for a calibration dataset for each estimation type.

EstimationType	$f_{x}$ (N)	$f_{y}$ (N)	$f_{z}$ (N)	$\tau_{x}$ (N m)	$\tau_{y}$ (N m)	$\tau_{z}$ (N m)
SnTdTO	55.4488	4.7026	−24.7221	−0.0345	−0.3811	0.5427
SwTdTO	60.0955	4.8701	−35.1782	0.0944	−0.5585	0.5305
SwTrTO	58.8686	5.5569	−42.5960	0.1131	−0.4363	0.5453
CnTdTO	55.1129	4.7137	−24.2897	−0.0422	−0.3659	0.5439
CwTdTO	59.1127	8.4534	−83.3496	0.3281	−0.1478	0.5903
CwTrTO	56.5174	6.0268	−45.0282	0.0878	−0.2893	0.5602
OnTdTO	55.1129	4.7137	−24.2897	−0.0422	−0.3659	0.5439
OwTdTO	59.1127	8.4534	−83.3496	0.3281	−0.1478	0.5903
OwTrTO	56.5174	6.0268	−45.0282	0.0878	−0.2893	0.5602

**Table 6:** Best calibration matrix by axis.

	Using Only Estimated C		Using Estimated C +o		
Axis	Best *C*	Value	Best *C*	Value	Workbench
$f_{x}$	SuppOnly λ5 OnTdTO	3.03893 N	All General λ5000 OnTdTO	3.14859 N	8.9007 N
$f_{y}$	SuppOnly λ1000 SwTdTO	2.42722 N	All General λ10 OwTrTO	3.05630 N	11.1776 N
$f_{z}$	SuppOnly λ10 OwTrTO	2.61958 N	SuppOnly λ1000 OwTrTO	2.40174 N	3.9954 N
$\tau_{x}$	SuppOnly λ1 SnTdTO	0.68899 Nm	All General λ100 OnTdTO	0.58208 Nm	0.7901 Nm
$\tau_{y}$	SuppOnly λ10000 OnTdTO	0.48474 Nm	SuppOnly λ100000 OnTdTO	0.43218 Nm	0.7146 Nm
$\tau_{z}$	SuppOnly λ100000 SnTdTO	0.15184 Nm	All General λ100 OwTdTO	0.18044 Nm	0.2769 Nm

**Table 7:** Offset estimated while hanging using IMU.

		Contact Forces			Error		
Support	In Situ	$F_{x}$ (N m)	$F_{y}$ (N m)	$F_{z}$ (N m)	$E_{x}$ (N m)	$E_{y}$ (N m)	$E_{z}$ (N m)
Double	No	−14.0252	−6.8170	342.5232	−14.0252	−6.8170	17.8122
Double	Yes	−5.5024	0.4523	324.2421	−5.5024	0.4523	−0.4689
Left	No	−17.5327	18.1905	342.6850	−17.5327	18.1905	17.9740
Left	Yes	−2.4991	−0.6910	325.3190	−2.4991	−0.6910	0.6080
Right	No	−28.3467	−9.4651	343.5918	−28.3467	−9.4651	18.8808
Right	Yes	−8.3183	−0.0448	323.8349	−8.3183	−0.0448	−0.8761

**Table 8:** Offset estimated while on left foot imposing mass.

		Contact Forces			Error		
Support	In Situ	$F_{x}$ (N m)	$F_{y}$ (N m)	$F_{z}$ (N m)	$E_{x}$ (N m)	$E_{y}$ (N m)	$E_{z}$ (N m)
Left	No	0.0733	0.0390	323.9000	0.0733	0.0390	−0.8110
Left	Yes	0.0119	0.0543	324.2001	0.0119	0.0543	−0.5109
Double	No	−4.3180	−31.3537	324.2382	−4.3180	−31.3537	−0.4728
Double	Yes	−8.1497	−5.3398	326.7298	−8.1497	−5.3398	2.0188
Right	No	−36.9491	−21.6199	343.4446	−36.9491	−21.6199	18.7336
Right	Yes	7.6190	−7.2226	321.7694	7.6190	−7.2226	−2.9416

## Supplementary Materials

The following are available online at https://www.mdpi.com/1424-8220/19/24/5521/s1, Video S1: SensorInsitu Video.

## Appendix A. Contact Forces Estimation Table Results

**Table A1:** Average magnitude of the contact force using only estimated calibration matrices.

		λ													Total	
Data Set	Estimation Type	0	1	5	10	50	100	1000	5000	10,000	50,000	100,000	500,000	1,000,000	By Type	By Dataset
	SnTdTO	14.7353	14.7229	14.6936	14.6531	14.3588	14.0140	10.5665	12.1406	13.3500	13.4198	12.9258	12.1937	12.0329	173.8069	
	SwTdTO	20.8714	20.8546	20.8302	20.7792	20.4633	20.0729	15.2905	11.1392	11.5100	12.2514	12.0020	11.6935	11.8216	209.5798	
	SwTrTO	8.0631	8.0634	8.0598	8.0505	8.0048	7.9535	8.0022	9.6557	10.5474	11.7435	11.8696	11.8197	11.8223	123.6554	
noTz	OnTdTO	21.4168	21.4005	21.3597	21.2985	20.8185	20.2611	13.5881	10.7362	12.2508	12.5915	12.0330	11.4229	11.5627	210.7403	
	OwTdTO	6.8583	6.8588	6.8500	6.8391	6.7752	6.7267	7.5632	10.5579	11.5182	11.8767	11.7000	11.3755	11.5138	117.0134	
	OwTrTO	6.8583	6.8588	6.8500	6.8391	6.7752	6.7267	7.5632	10.5579	11.5182	11.8767	11.7000	11.3755	11.5138	117.0134	951.8093
	SnTdTO	8.9899	8.9870	8.9852	8.9788	8.9340	8.8807	8.1122	7.0044	7.0709	8.7356	9.3448	10.1252	10.3787	114.5273	
	SwTdTO	7.8568	7.8522	7.8563	7.8474	7.8220	7.7810	7.2966	6.7480	6.9413	8.5509	9.1050	9.8410	10.2381	105.7365	
	SwTrTO	5.9704	5.9744	5.9739	5.9719	5.9617	5.9476	5.8722	6.2594	6.8156	8.6707	9.2587	10.0058	10.2779	92.9601	
suppOnly	OnTdTO	8.4813	8.4812	8.4742	8.4694	8.4277	8.3757	7.6485	6.5679	6.5622	7.9906	8.5353	9.3383	9.8301	107.1824	
	OwTdTO	5.5253	5.5224	5.5242	5.5197	5.5159	5.5066	**5.4980**	6.0596	6.6191	8.1749	8.6419	9.3260	9.7944	87.2280	
	OwTrTO	5.5253	5.5224	5.5242	5.5197	5.5159	5.5066	**5.4980**	6.0596	6.6191	8.1749	8.6419	9.3260	9.7944	87.2280	594.8624
	SnTdTO	8.8400	8.8376	8.8288	8.8280	8.7825	8.7298	7.9563	6.7394	6.7075	8.4183	9.1163	10.0199	10.3114	112.1158	
	SwTdTO	7.6831	7.6772	7.6757	7.6696	7.6372	7.6019	7.1228	6.5264	6.6573	8.3207	8.9462	9.8235	10.2860	103.6277	
	SwTrTO	5.9078	5.9070	5.9082	5.9047	5.8935	5.8812	5.7327	5.9845	6.4650	8.3763	9.0411	9.9018	10.2382	91.1419	
All	OnTdTO	8.3123	8.3180	8.3107	8.3098	8.2603	8.2133	7.4991	6.3815	6.3095	7.7947	8.4078	9.3503	9.8907	105.3580	
	OwTdTO	5.6319	5.6321	5.6326	5.6320	5.6240	5.6056	5.5185	5.9089	6.4084	7.9956	8.5170	9.3321	9.8544	87.2930	
	OwTrTO	5.6319	5.6321	5.6326	5.6320	5.6240	5.6056	5.5185	5.9089	6.4084	7.9956	8.5170	9.3321	9.8544	87.2930	586.8295
Total By λ		163.1593	163.1028	162.9699	162.7426	161.1946	159.3902	141.8470	140.9359	150.2790	172.9581	178.3033	185.6028	191.0157		
Workbench		16.6642	16.6642	16.6642	16.6642	16.6642	16.6642	16.6642	16.6642	16.6642	16.6642	16.6642	16.6642	16.6642		

**Table A2:** Average magnitude of the contact force using also estimated Offset.

		λ													Total	
Data Set	Estimation Type	0	1	5	10	50	100	1000	5000	10,000	50,000	100,000	500,000	1,000,000	By Type	By Dataset
	SnTdTO	14.761	14.751	14.725	14.689	14.426	14.127	11.224	13.534	14.833	14.739	14.207	13.434	13.179	182.63	
	SwTdTO	21.386	21.367	21.349	21.305	21.053	20.725	17.116	14.779	14.856	14.452	14.022	13.594	13.671	229.68	
	SwTrTO	8.7331	8.7367	8.7304	8.7365	8.7305	8.7276	9.1473	10.916	11.609	11.811	11.597	11.059	10.887	129.42	
noTz	OnTdTO	21.959	21.944	21.912	21.859	21.439	20.958	15.469	13.667	14.719	14.673	14.182	13.785	13.973	230.54	
	OwTdTO	7.469	7.4694	7.4693	7.454	7.4014	7.3759	8.1999	10.817	11.619	11.667	11.393	10.94	11.026	120.3	
	OwTrTO	7.469	7.4694	7.4693	7.454	7.4014	7.3759	8.1999	10.817	11.619	11.667	11.393	10.94	11.026	120.3	1012.9
	SnTdTO	9.4503	9.4505	9.4492	9.4439	9.4308	9.3986	9.1777	9.4486	10.032	11.329	11.628	11.967	11.943	132.15	
	SwTdTO	10.132	10.132	10.131	10.124	10.131	10.144	10.313	10.951	11.404	12.072	12.181	12.458	12.6	142.77	
	SwTrTO	7.0649	7.0602	7.0611	7.0615	7.0675	7.0794	7.2527	7.919	8.2931	8.8514	8.9516	9.0766	9.0849	101.82	
suppOnly	OnTdTO	9.9833	9.9845	9.9838	9.9833	9.9844	9.9951	10.069	10.639	11.161	12.163	12.418	12.9	13.14	142.4	
	OwTdTO	6.0123	6.0116	6.0139	6.0117	6.0181	6.026	6.2234	7.0101	7.4735	8.3683	8.6422	9.1567	9.4101	92.378	
	OwTrTO	6.0123	6.0116	6.0139	6.0117	6.0181	6.026	6.2234	7.0101	7.4735	8.3683	8.6422	9.1567	9.41	92.378	703.91
	SnTdTO	9.3042	9.3057	9.298	9.296	9.2784	9.2545	8.9752	9.071	9.5762	10.919	11.254	11.654	11.691	128.88	
	SwTdTO	9.9729	9.9645	9.9677	9.9716	9.9789	9.9759	10.093	10.658	11.074	11.782	11.918	12.228	12.421	140.01	
	SwTrTO	6.9187	6.9182	6.9185	6.9183	6.9151	6.9269	6.9783	7.4763	7.8228	8.4421	8.5773	8.7733	8.8471	98.433	
All	OnTdTO	9.712	9.722	9.7206	9.7194	9.7138	9.722	9.7785	10.278	10.791	11.868	12.14	12.667	12.97	138.8	
	OwTdTO	5.8685	5.868	5.8659	**5.8647**	5.8708	5.8657	5.9457	6.5411	6.99	8.0151	8.3514	8.9122	9.227	89.186	
	OwTrTO	5.8685	5.868	5.8659	**5.8647**	5.8708	5.8657	5.9457	6.5411	6.99	8.0151	8.3514	8.9122	9.227	89.186	684.49
Total		178.08	178.03	177.94	177.77	176.73	175.57	166.33	178.07	188.34	199.2	199.85	201.61	203.73		
Workbench		16.6642	16.6642	16.6642	16.6642	16.6642	16.6642	16.6642	16.6642	16.6642	16.6642	16.6642	16.6642	16.6642		
